# Bound to a spider without its web: Task-type modulates the retrieval of affective information in subsequent responses

**DOI:** 10.3758/s13414-023-02791-5

**Published:** 2023-10-18

**Authors:** Lars-Michael Schöpper, Alicia Jerusalem, Lisann Lötzke, Christian Frings

**Affiliations:** 1 Department of Cognitive Psychology, University of Trier, Trier, Germany; 2 Department of Psychology, Friedrich-Schiller University Jena, Jena, Germany; 3 Institute for Cognitive and Affective Neuroscience (ICAN), University of Trier, Trier, Germany

**Keywords:** Attention, Action control, S-R binding, Inhibition of return, Approach-avoidance, Task dependency

## Abstract

Action control theories assume that upon responding to a stimulus response and stimulus features are integrated into a short episodic memory trace; repeating any component spurs on retrieval, affecting subsequent performance. The resulting so-called “binding effects” are reliably observed in discrimination tasks. In contrast, in localization performance, these effects are absent and only inhibition of return (IOR) emerges – a location change benefit. Affective information has been found to modulate binding effects; yet a modulation of IOR has led to mixed results, with many finding no influence at all. In the current study, participants discriminated letters (Experiment 1) or localized dots (Experiment 2) on a touchpad in prime-probe sequences. During the prime display two images – one with fruits and one with a spider – appeared, one of which spatially congruent with the to-be-touched area. In the discrimination task, previously touching a spider compared to a fruit slowed down response repetitions. In contrast, the localization task only showed IOR. This suggests that task-irrelevant valence is integrated with the response and affects subsequent responses due to retrieval. However, this is not ubiquitous but depends on task type. The results shed further light on the impact of affective information on actions.

## Introduction

Imagine you have to reach for a certain cable behind your desk. While initiating the reaching movement, you notice spider webs and a huge spider sitting next to the cable you are aiming to grab. The spider is not the cause of your action, but close or even direct contact is still unavoidable. Will the spider hinder your actions, even if it has already hidden startled by your previous movements? Humans are highly sensitive to threat detection (e.g., Lang et al., 2000; Öhman & Soares, 1993; Öhman et al., 2001). In turn, bodily movements have been found to be modulated by the aversiveness of certain stimuli, for example, in that participants approach positive but avoid negative stimuli (e.g., Chen & Bargh, 1999; Eder & Rothermund, 2008; Elliot, 2006; Phaf et al., 2014). This can also have an impact on performance in experimental tasks when affective information is spatially congruent with the response (Yamaguchi et al., 2018), if positive and negative valence of stimuli is directly mapped to a response (Yamaguchi & Chen, 2019; see also Blask et al., 2016, and Proctor, 2013), or if affective information is presented (on the handle of) graspable objects (Scerrati et al., 2022). Further, grasping objects of positive and negative valence is differently affected by using the dominant versus non-dominant hand (Michalland et al., 2019). In short: Valence has the power to affect behavior.

## Action control and affect

According to binding approaches in action control, when responding to a stimulus with certain features, stimulus features and the response are integrated into a short episodic memory trace, referred to as an event file (Frings et al., 2020; Hommel, 2004). If any information of the previous event file repeats – for example, a non-spatial feature (e.g., color or shape), the location, or the response – the previous event file is retrieved and affects performance. This is typically measured in prime-probe sequences, in which participants respond to a target in a prime display followed by the response to a target in a probe display (e.g., Frings et al., 2007). From prime to probe, response-relevant and response-irrelevant features are systematically varied to repeat or change, like indicating the shape of a stimulus (response-relevant feature) while repeating or changing its response-irrelevant color (e.g., Singh et al., 2016). Repeating both the response and response-irrelevant features from prime to probe is beneficial for responding as the previous event file can be fully retrieved. However, if the response repeats, but the response-irrelevant feature changes, interference occurs (e.g., Schöpper, Singh, & Frings, 2020; Singh et al., 2016); similar, if the response changes, but the response-irrelevant feature repeats, said feature retrieves the previous event file, slowing down responding and increasing error rates compared to full repetitions. Lastly, changing all information from prime to probe, nothing is retrieved – so neither a retrieval-induced benefit nor interference occurs. These partial repetition costs (Hommel, 1998, 2004) caused by binding and retrieval (Frings et al., 2020) lead to so-called stimulus-response (S-R) binding effects and can be found when discriminating color (Laub et al., 2018; Schöpper, Hilchey et al., 2020), shape (Schöpper, Singh, & Frings, 2020; Singh et al., 2016), letters (Frings et al., 2007; Schmalbrock et al., 2022), pitch (Moeller et al., 2012), and so on. Furthermore, S-R binding explanations exist for a number of experimental paradigms with sequential designs as priming (Henson et al., 2014), task-switching (Koch et al., 2018), conflict tasks (Davelaar & Stevens, 2009), and more (for an overview, see Frings et al., 2020).

Previous research has found that valence can have an impact on S-R binding effects, when affective images are presented in-between targets (Colzato et al., 2007), when using words matching or not-matching in their valence (Giesen & Rothermund, 2011), or when instructing participants to attend to otherwise task-irrelevant valence of words (Singh et al., 2018). Congruent with the latter, faces with affective expressions only modulate binding and retrieval if task-relevant or under specific conditions (Coll & Grandjean, 2016; Coll et al., 2019) but not if completely task-irrelevant (see also Trübutschek & Egner, 2012). Moreover, affect in action control paradigms can be investigated as a consequence of effect anticipation of certain actions. According to this view (see also Lavender & Hommel, 2007), affect can become integrated with the action that led to it (e.g., Eder & Hommel, 2013; Eder & Klauer, 2009; Eder et al., 2015, 2017). These emotional effects – even if completely task-irrelevant – can have an influence on action control processes (Eder et al., 2015) congruent with S-R binding assumptions (see Frings et al., 2020).

To conclude, S-R binding approaches in action control assume that response and features can be integrated into a common representation (Hommel, 1998, 2004) and that upon repetition of any of its components – response and/or features – the previous information is retrieved, affecting performance (Frings et al., 2020). Affect as a non-spatial feature has been found to have an impact on task executions congruent with S-R binding assumptions (e.g., Colzato et al., 2007; Eder et al., 2015; Giesen & Rothermund, 2011; see also Frings et al., 2020).

## Attentional orienting and affect

Although to be assumed to underlie all actions, that is, a bodily movement with an intention in mind (e.g., Frings et al., 2020), S-R binding is not as ubiquitous as previously thought. If participants have to signal the detection or location of stimuli sequentially appearing somewhere, a benefit for location changes arises, that is, inhibition of return (IOR; Posner et al., 1985; for a review, see, e.g., Klein, 2000), typically unmodulated by repeating or changing non-spatial features (color, shape, and so on). This is in stark contrast to what would be predicted by action control theories (e.g., Huffman et al., 2018; Schöpper & Frings, 2022, 2023; Schöpper, Hilchey et al., 2020; however, see Schöpper & Frings, 2023, for binding effects in auditory detection performance). As the trial sequences can be identical (see, e.g., Huffman et al., 2018; Schöpper & Frings, 2022; Schöpper, Hilchey et al., 2020, that is, responding to two stimuli in a sequence, the emerging differences in observed S-R binding effects are attributed to task demands that are present in discrimination tasks but absent in detection and localization performance. For example, it is thought that the latter two types of task lack attention towards task-irrelevant features (Huffman et al., 2020; see also Hommel, 2007; Hommel et al., 2014), and/or lack post-selective processing after target identification (Schöpper & Frings, 2022; Schöpper et al., 2022a, b; see also Zehetleitner et al., 2012).

However, there are a few occasions of non-spatial feature repetitions and changes affecting performance in non-discrimination tasks. So-called non-spatial IOR (Law et al., 1995) refers to slower responding if the non-spatial feature of a target repeats (see, however, Fox & de Fockert, 2001, arguing that this pattern is better explained by repetition blindness), especially pronounced at location repetitions (Hu et al., 2011, 2013). More relevant to the current study, several researchers have aimed at finding modulations of valence on IOR; for this, traditionally the cue-target paradigm has been used. Here, a cue appears at a location followed by the target appearing at the same or a different location; participants are instructed to signal the detection (Posner & Cohen, 1984) or location (Taylor & Klein, 2000) of the target. Under quite specific experimental conditions (e.g., subliminal presentation, Pan et al., 2017; blocked presentation, Rutherford & Raymond, 2010; keeping the location constant, Chao, 2010; assigning learned loss and gain values to cues, Rutherford et al., 2010; investigating anxious participants, Broomfield & Turpin, 2005; using angry versus happy or neutral faces as stimuli, Fox et al., 2002), effects have been found in detection and localization procedures. However, multiple studies have shown that when positive and negative images (e.g., spiders or fearful faces as stimuli) are used as cues (Berdica et al., 2017; Lange et al., 2008; Stoyanova et al., 2007) or as targets (Berdica et al., 2017; Silvert & Funes, 2016, Experiment 1) a modulation of detection or localization responses is typically absent. In contrast – and especially interesting in the context of the presence of S-R binding in discrimination procedures (Schöpper, Hilchey et al., 2020) – when the response to the target following the cue is a discrimination response, a modulation by valence can be regularly observed (Perez-Duenas et al., 2014; Silvert & Funes, 2016, Experiments 3 and 4; Yiend & Mathews, 2001).

## The current study

In the current study, we aimed to investigate if task-irrelevant valence can become bound to a response and become retrieved by simply repeating the response. We did so by having participants respond to prime-probe sequences on a touchpad. In typical prime-probe sequences investigating binding effects, a task-irrelevant feature is varied to repeat or change from prime to probe (e.g., color; Schöpper & Frings, 2022). However, as we were specifically interested in if affective information is bound to the response and retrieved by the latter (and not if affective information retrieves a previous response), task-irrelevant valence was only presented in the prime display. Thus, during the prime display, an image of a fruit (or a fruit arrangement) and an image of a spider was shown; one image was presented at one of the two response fields on the monitor for the target presented above, respectively. By that, participants had to execute the response to the prime target on a field at which either fruits (positive stimulus) or a spider (negative stimulus) were presented. There were no affective images in the probe display. If task-irrelevant information is integrated with the response and affects subsequent responses due to retrieval (e.g., Frings et al., 2020; Hommel, 2004) and if this also includes the coupling of affective information (e.g., Giesen & Rothermund, 2011; Singh et al., 2018; cf. Yamaguchi & Chen, 2019), task-irrelevant valence should be integrated with the prime response – and should interfere with response executions to the probe target. Response repetitions should suffer from previously touching a spider image compared to a fruit image, due to the response repetition retrieving the aversiveness of the previous action. In contrast, response changes should show no difference in reaction times due to previously responding on a spider or fruit image, as nothing is retrieved.^1^ If the effect can be explained via binding and retrieval of affective information, this pattern should only be observed when *discriminating* a target stimulus, but not when *localizing* a target stimulus (cf. Schöpper & Frings, 2022). For that matter, we led participants discriminate target letters (Experiment 1) or localize target dots (Experiment 2) while otherwise using identical experimental procedures. In turn, for the localization task we only expect to observe IOR unmodulated by previous valence (cf. Berdica et al., 2017), as IOR being unmodulated by non-spatial information is the common pattern in said task type (Huffman et al., 2018; Schöpper & Frings, 2022; cf., Experiment 2 in Schöpper et al., 2022a).

Of note, localization procedures often come with the limitation that response and location are completely confounded, that is, a response repetition and change always indicate a location repetition and change (see, e.g., Hilchey et al., 2019; Huffman et al., 2018; Schöpper et al., 2022a; Schöpper & Frings, 2022). In contrast, in non-spatial discrimination procedures it is possible to disentangle stimulus repetitions and changes from response repetitions and changes, for example, by using more than one non-spatial feature mapped to the response. Resulting binding effects might vary on repeating versus changing a target stimulus in response repetitions (e.g., Giesen & Rothermund, 2014), but do not necessarily do so (e.g., Schöpper, Singh, & Frings 2020; Singh et al., 2016). In both experiments of the current study, we only used one (non-)spatial feature for each response. By that, target and response are completely confounded in discrimination (as, e.g., the color discrimination tasks in Schöpper, Hilchey et al., 2020) and localization (Schöpper & Frings, 2022) performance. Thus, any effect can be derived from the discrimination versus localization response, that is, a response based on post-selective processing of a target (cf., Schöpper, Hilchey et al., 2020; Zehetleitner et al., 2012) versus a response that can be executed based on direct identification (e.g., Huffman et al., 2018; Schöpper & Frings, 2022; Schöpper et al., 2022a).

## Methods

The experimental procedure for Experiment 1 was preregistered at https://aspredicted.org/kr4yg.pdf. We did not separately preregister Experiment 2, however, the procedure was mostly identical to Experiment 1, unless stated otherwise.

## Experiment 1: Discrimination task

### Participants

For sample size estimation we referred to previous studies involving the binding of task-irrelevant features. Here, binding effects are usually stable and strong with medium to high effect sizes (Cohen’s *d* between 0.4 and 0.8; e.g., Frings et al., 2007; Schöpper, Singh, & Frings, 2020). In order to find a medium-sized binding effect with an expected effect size of *d* = 0.5, the experiment was run with *N* = 30 participants, which – assuming α = .05 (one-tailed) – results in a power of 1 - β = 0.85 (G * Power, Version 3.1.9.2; Faul et al., 2007). Thirty students of the University of Trier (26 women, four men, *M*_age_ = 23.9, *SD*_age_ = 3.4 years, age range: 19–34 years) participated for either partial course credit or a monetary reward of 5€. Prior to experiment start, participants were informed that the study involved the presentation of spider images; all participants gave written informed consent. Two participants reported some minor uncorrected visual impairments, but their data was inconspicuous when compared with the sample. All other participants reported normal or correct-to-normal vision.

### Apparatus and materials

The experiment was conducted with E-Prime 2.0. The experiment was primarily run on a touchpad (7-in. touch monitor, Faytech Ldt., Henzen, China). The instructions appeared on a monitor positioned behind the touchpad prior each task; this screen turned black during the experiment. The touchpad was mounted to a stand and could be grasped with the left and right hand. From an approximate distance of 45 cm, the screen of the touchpad encompassed an area of 19.42° x 10.92° of visual angle on which a resolution of 1,920 x 1,080 pixels was displayed. All stimuli appeared on a black background. A white fixation cross (0.51 ° x 0.51°) was presented in the center of the upper area of the screen. The two targets were the letters ‘M’ or ‘W’, measuring 0.89° x 0.89°, written in Courier New, and appeared at the same position as the fixation cross. In the lower part of the screen and to left and right below the fixation cross, two response fields appeared constantly across all trials. These fields were marked by rectangular white frames with 0.13° line thickness, spanning an area of approximately 7.12° x 5.34°. These were either unfilled (i.e., black) or filled with affective stimulus material, that is, photographs, measuring a 6.87° x 5.09°.

As negative affective stimuli, we used 53 photographs^2^ of different spiders. These were selected from the 'Geneva affective picture database' (GAPED; Dan-Glauser & Scherer, 2011) and depicted different species of spiders on various bases (e.g., walls, plants, wood, etc.). As positive affective stimuli, we used 53 photographs of fruits. Photographs were taken in our lab and consisted of a variable set of different types of berries – raspberries, blueberries, blackberries, and grapes presented in different arrangements – positioned on various bases (e.g., yellow plate, wooden board, red-white checkered towel, etc.). The set of fruit stimuli is available upon request.

The diagonal distance between fixation cross/target letter and the response field/image was 7.5° (center-to-center). The two response fields where the affective stimulus material was presented were approximately 10.66° (center-to-center) apart. Responses to the target stimulus 'M' were made to the left response field, whereas responses to the target stimulus 'W' were made to the right response field of the touchpad by pressing them with the left or right thumb, respectively. A response was logged as a left or right response if it was given to the left or right screen-half, respectively. A response was only logged if it was executed in the lower area of the screen, that is, below 0.21° above the upper line of each response field. Note that the area for responses on the touchpad was slightly larger than the visual representation of response fields themselves; however, response coordinates were logged to control for participants avoiding to click the response fields.

For exploratively investigating the impact of fear of spiders on the effects of interest, we included the ‘Fragebogen zur Angst vor Spinnen’ (FAS; Rinck et al., 2002), the German translation of the ‘Fear of Spiders Questionnaire’ (FSQ; Szymanski & O'Donohue, 1995), an 18-item self-report questionnaire with a duration of approximately 5 min. Participants indicated their agreement with 18 statements (e.g., 'If I saw a spider now, I would think it will harm me') on a 7-point Likert scale, ranging from 0 to 6. A higher value indicates higher fear of spiders. Each statement was presented on the touchscreen and participants gave their responses with their right index finger directly below on quadratic-shaped and horizontally-aligned response fields labelled with numbers ranging from 0 to 6.

### Design

The experiment used a 2 (response relation: repetition vs. change) x 2 (prime valence mapping: positive vs. negative) repeated-measures design. The two independent variables were varied within-subjects. The hypothesis-relevant effect was expected to be a significant interaction of response relation and valence mapping.

### Procedure

Participants were tested individually in single sessions. Before the start of the experiment, participants were informed that the experiment involved the presentation of pictures of spiders. The instructions appeared on a monitor behind the touchpad. The instructions included a photograph of how to position the hands. The participants grasped the touchpad with the left and right hand so that the required responses on the response fields on the screen could be given using their thumbs. After the last instructions-slide, this screen turned black and the experiment started on the touchpad. Within a sequence, participants discriminated two target stimuli – left thumb response for letter M and right thumb response for letter W.

**Fig. 1:**
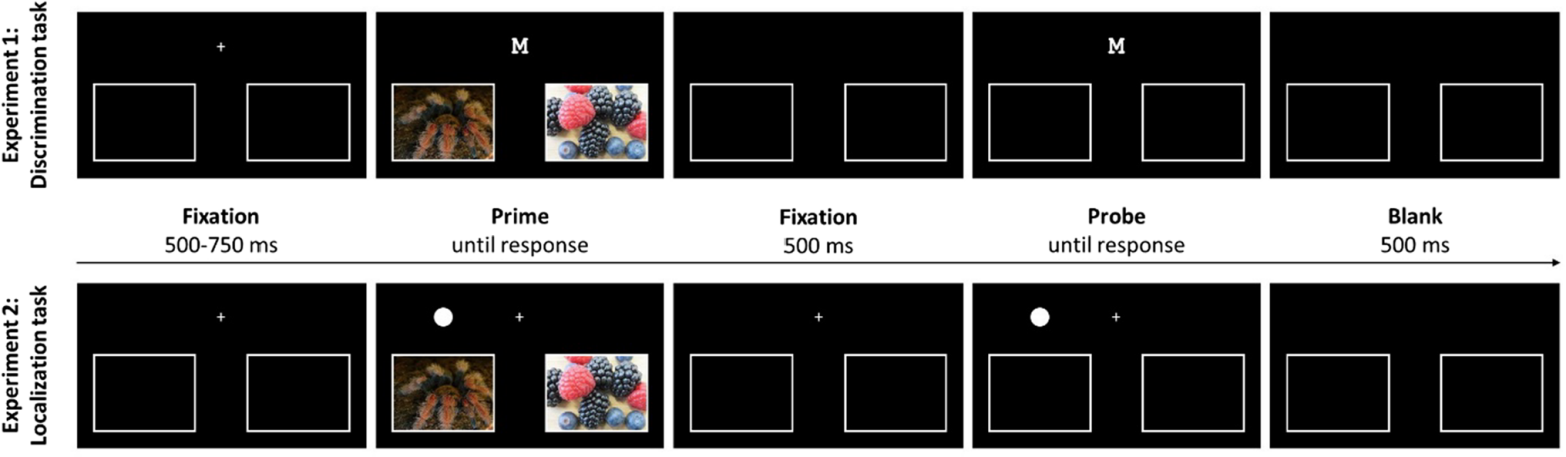
An exemplary trial sequence of the discrimination task (Experiment 1) and the localization task (Experiment 2). Participants classified the letter (top) or localized the dot (bottom) via keypress to the left frame (containing a spider in the prime but no picture in the probe). Both sequences depict a trial in which the response repeats with a negative mapping during the prime display (i.e., response repetition with negative mapping, RRN). (Spider image ID: SP052 in Dan-Glauser & Scherer, 2011)

The experiment used prime-probe sequences. In the prime display, participants responded to a first target stimulus, followed by a probe display with a response to the second target stimulus. A trial started with a fixation cross in the upper part of the screen with a variable presentation duration of 500–750 ms. Participants were instructed to fixate this area throughout presentation. In the following prime display, a target letter – M or W – replaced the fixation cross. Crucially, during the prime display, two affective pictures of different valence appeared in the two response fields (see Fig. 1). Participants were instructed to respond to the letter M by pressing the left response field (with the left thumb), and by pressing the right response field (with the right thumb) to the letter W. The prime display was presented until a response was given. As soon as participants gave their response on one of the response fields filled with affective stimulus material, the letters and affective stimuli disappeared, leaving only the frames of the two response fields visible for 500 ms. This was followed by the presentation of the probe stimulus, to which the discrimination response was made as described for the prime display. However, in contrast to the prime display, no affective images appeared in the response fields during the probe display. Participants simply gave their responses to the outlined fields in the lower part of the screen. After incorrect responses, an error notification appeared for 1,500 ms. After the probe response, the target letter disappeared, leaving the upper area blank for 500 ms, ending one prime-probe sequence. If participants responded incorrectly during prime or probe display, an error message appeared in the upper part of the screen for 1,500 ms directly following the incorrect response. Figure 1, upper row, depicts an example of a prime-probe sequence in Experiment 1.

The experiment started with a total of 16 practice trials, followed by 320 experimental trials (the first half of experimental trials was labelled as a “training”-phase, followed by the “experiment”-phase; however, labelling was done to make two blocks). Trial conditions were response repetition with positive valence mapping in the prime display (RRP), response repetition with negative valence mapping in the prime display (RRN), response change with positive valence mapping in the prime display (RCP), and response change with negative valence mapping in the prime display (RCN). The practice phase consisted of four trials for each condition, followed by the experimental trials consisting of 80 prime-probe sequences for each condition. Trial conditions were presented randomly. Each of the 53 spider and each of the 53 fruit pictures was randomly presented six times during the experimental trials (i.e., in 318 trials; with two positive and two negative pictures presented a seventh time each to reach a total of 320 trials). The position of the positive and negative picture pair as left/right or right/left varied randomly from trial to trial and was orthogonally varied with response repetitions and changes. During practice trials, 16 images were drawn randomly from each stimulus set. After the 160th experimental trial, there was a short break, which participants terminated by their own choice. After completing the experimental trials, the experiment concluded with participants working through the 18-item FAS.

## Results

### Reaction times

Probe reaction times below 200 ms or above 1.5 interquartile range above the third quartile of a participant’s distribution (Tukey, 1977) were excluded from analysis. Additionally, trials were only included for analysis, if both prime response and probe response were correct. Due to these constraints, 13.51% of probe trials were discarded. In our analysis, we deviate from our pre-registered plan in two occasions, that is, by using a more conservative cut-off criterium for reaction times and by analyzing both blocks (i.e., training and experiment trials). We elaborate this decision further in Appendix 1.

**Fig. 2:**
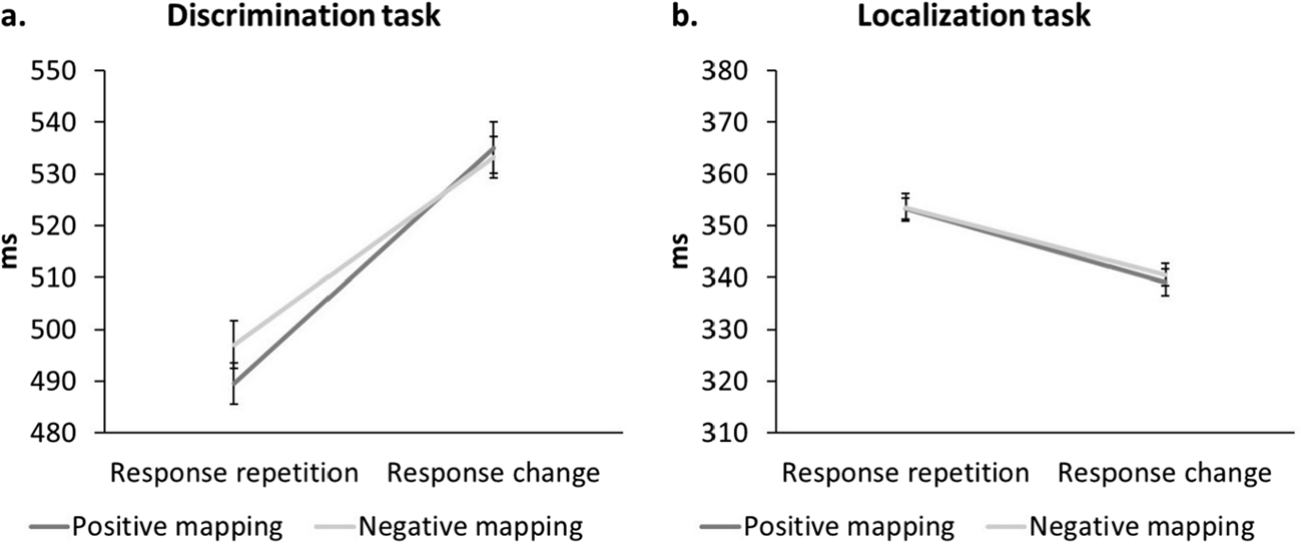
The interaction of response relation and valence mapping of (**a**) the discrimination task (Experiment 1), and (**b**) the localization task (Experiment 2). Error bars represent the within-subject standard error after Cousineau (2005) with correction by Morey (2008)

A 2 (response relation: repetition vs. change) x 2 (prime valence mapping: positive vs. negative) repeated-measures ANOVA revealed a significant main effect of response relation, *F*(1, 29) = 49.67, *p* < .001, ${\upeta }_{p}^{2}$ = .63. Participants responded faster if the response repeated (493 ms) compared to changed (534 ms). The main effect of valence mapping was not significant, *F*(1, 29) = 1.07, *p* = .310, ${\upeta }_{p}^{2}$ = .04. Crucially, the interaction between response relation and valence mapping turned significant, *F*(1, 29) = 6. 51, *p* = .016, ${\upeta }_{p}^{2}$ = .18. In response repetition trials, participants were faster if they previously had given a response on a positive image (490 ms) compared to a negative image (497 ms). This pattern was slightly reversed for response changes: In these, participants responded faster when they previously had pressed on a negative image (533 ms) compared to a positive image (535 ms; see Fig. 2a). Post hoc *t*-tests showed that the difference in response repetitions was significant, *t*(29) = 2.48, *p* = .019, *d* = 0.45, whereas that in response changes was not, *t*(29) = 0.50, *p* = .622, *d* = 0.09.

To ease interpretation, we recalculated the interaction into a differential value by (RRN-RRP)-(RCN-RCP). This value sums up the benefit of previously responding to a positive image for response repetitions with the benefit of previously responding to a negative image for response changes. This differential value was 9 ms, significant when tested against 0, *t*(29) = 2.55, *p* = .016, *d* = 0.47, and had a *BF*_10_ = 3.00 (Cauchy prior = 0.707 in JASP; JASP Team, 2023) in favor of the alternative hypothesis given the data.

### Error rates

Error rate is the percentage of incorrect probe responses to correct probe responses after the prime response was correct. In turn, probe trials were only included for analysis, if the previous prime response was correct. Due to this constraint, 4.17% of probe trials were excluded from the analysis.

A 2 (response relation: repetition vs. change) x 2 (valence mapping: positive vs. negative) repeated-measures ANOVA on probe error rates revealed a significant main effect of response relation, *F*(1, 29) = 12.66, *p* = .001, ${\upeta }_{p}^{2}$ = .30, in that participants made more errors when the response changed (4.73%) compared to repeated (2.81%). There was no main effect of valence mapping, *F*(1, 29) = 1.37, *p* = .252, ${\upeta }_{p}^{2}$ = .05. The interaction of response relation and valence mapping was not significant, *F*(1, 29) = 0.41, *p* = .530, ${\upeta }_{p}^{2}$ = .01 (RRP: 2.96%; RRN: 2.66%; RCP: 5.08%; RCN: 4.39%). The differential value was 0.40%, not significant when tested against 0, *t*(29) = 0.64, *p* = .530, *d* = 0.12, and had a *BF*_01_ = 4.27 in favor of the Null hypothesis.

### Explorative analysis

First, we exploratively looked at the impact of spider fear on task performance, as often a pronounced effect for spider-fearful individuals is observed (e.g., Mogg & Bradley, 2006; Rinck & Becker, 2007; Rinck et al., 2016). From the 18-item FAS (Rinck et al., 2002; German translation of FSQ, Szymanski & O'Donohue, 1995), we calculated a mean score for every participant, which could range between 0 and 6. The overall mean across all participants was *M*_FAS_ = 1.33, *SD*_FAS_ = 1.28, and the questionnaire’s reliability was high (Cronbach’s α = .96). We correlated FAS with the differential values of the interactions for reaction times and error rates; FAS did not significantly correlate with the differential value for reaction times (*r* = -.17, *p* = .359) nor error rates (*r* = .11, *p* = .560). Hence, increased fear of spiders did not affect the interactions in reaction times or error rates, and does not appear to accentuate the impact of valence in subsequent responses.

Second, we looked at the effect over time, as showing many different spider images over the course of the experiment might have led to effects of habituation (cf. Matthews et al., 2012; Rowe and Craske, 1998). For doing so, we entered first and second block (i.e., training and experiment trials) as a within-subject factor to the ANOVA. For reaction times, this analysis revealed a main effect of block, *F*(1, 29) = 16.31, *p* < .001, ${\upeta }_{p}^{2}$ = .36, with faster responses in the second (507 ms) compared to first (521 ms) block. However, block did not modulate any other effects (all *F* ≤ 1.99, all *p* ≥ .170). For error rates, there was a three-way interaction of block, response relation, and valence mapping, *F*(1, 29) = 5.71, *p* = .024, ${\upeta }_{p}^{2}$ = .17 (all other effects *F* ≤ 2.53, *p* ≥ .122). To pinpoint this interaction, we looked at the blocks separately. In the first block, there was a main effect of response relation, *F*(1, 29) = 7.07, *p* = .013, ${\upeta }_{p}^{2}$ = .20 (RR: 2.91%; RC: 4.74%). The effect of valence mapping approached but did not reach significance, *F*(1, 29) = 2.98, *p* = .095, ${\upeta }_{p}^{2}$ = .09 (positive: 4.42%; negative: 3.23%). Crucially, the interaction turned significant, *F*(1, 29) = 5.39, *p* = .028, ${\upeta }_{p}^{2}$ = .16. For response repetitions, previously responding on a fruit (2.89%) compared to a spider (2.93%) did not change much; however, for response change previously responding on a fruit (5.95%) came with more errors than previously responding on a spider (3.52%). In the second block, only the main effect of response relation was significant, *F*(1, 29) = 10.52, *p* = .003, ${\upeta }_{p}^{2}$ = .27 (RR: 2.70%; RC: 4.72%; all other *F* ≤ 2.49, all *p* ≥ .125).

## Experiment 2: Localization task

### Methods

#### Participants

For Experiment 2, we used the same sample size as in Experiment 1. The classic IOR effect comes with a medium to large effect size, as well (e.g., Huffman et al., 2018; Schöpper, Hilchey et al., 2020). Thirty students (21 female, nine male, *M*_age_ = 23.0 *SD*_age _= 2.86 years, age range 18–30 years) from the University of Trier participated in exchange for partial course credit or a monetary reward of 5€. One participant reported a light color blindness, but the data was inconspicuous when compared with the sample. All other participants reported normal or corrected-to-normal vision.

### Apparatus, materials, design and procedure

Apparatus, materials, design, and procedure were identical to Experiment 1, except for the following. Instead of using letters repeating or changing their identity as in Experiment 1, Experiment 2 used a white dot (0.89° x 0.89°) repeating or changing its position. The target dot could appear in the left or right half of the screen, centrally above the left or right response field on the x-axis and in line with the fixation cross on the y-axis. Vertical distance between a response field and the target dot was 5.28° (center-to-center). Horizontal distance between fixation cross and target dot was 5.34° (center-to-center). Participants were instructed to localize the dot by touching the corresponding left or right response field on the touchpad. In contrast to Experiment 1, the fixation cross remained on screen during as well as in-between prime and probe displays (see Fig. 1, lower row).

## Results

### Reaction times

We used the same inclusion and upper cutoff criteria as in Experiment 1 except for lowering the lower cut-off to 100 ms due to overall faster responding. Due to these constraints, 7.34% of probe trials were discarded.

The 2 (response relation: repetition vs. change) x 2 (prime valence mapping: positive vs. negative) repeated-measures ANOVA on probe reaction times, revealed a main effect of response relation, *F*(1, 29) = 17.97, *p* < .001, ${\upeta }_{p}^{2}$ = .38. Participants were faster if the response – and by that the location – changed (340 ms), compared to when it repeated (353 ms), thus resembling IOR (e.g., Klein, 2000). The main effect of prime valence mapping was not significant, *F*(1, 29) = 0.66, *p* = .424, ${\upeta }_{p}^{2}$ = .02. Importantly, the hypothesis-relevant two-way interaction of response relation and prime valence mapping was not significant, *F*(1, 29) = 0.34, *p* = .567, ${\upeta }_{p}^{2}$ = .01. Response repetitions and changes were unaffected by previously responding on positive or negative images (RRP: 353 ms; RRN: 354 ms; RCP:339; RCN: 341 ms). The differential value of the interaction was -1 ms, not significant when tested against 0, *t*(29) = -0.58, *p* = .567, *d* = -0.11, and had a *BF*_01_ = 4.41 in favor of the null hypothesis given the data.

### Error rates

We excluded all trials in which the prime response was incorrect (0.97% of probe trials). Participants barely made errors (e.g., 15 participants had an error rate of 0% after a correct prime response). As error rates were very low, they could not be analyzed as reported for Experiment 1. In total (i.e., collapsed irrespective of condition), the overall error rate was 0.35%.

### Explorative analysis

As in Experiment 1, we calculated an average value of spider fear, resulting from the FAS. The overall mean of FAS across all participants was *M*_FAS_ = 0.76, *SD*_FAS_ = 0.82, and the questionnaire’s reliability was high (Cronbach’s α = .92). FAS was uncorrelated with the differential value of the interaction of response relation and valence mapping for reaction times, *r* = -.07, *p* = .720.

The block-order analysis revealed a main effect of block, *F*(1, 29) = 10.23, *p* = .003, ${\upeta }_{p}^{2}$ = .26, with faster responses in the second (339 ms) compared to the first (355 ms) block. The interaction of block and response relation approached significance, *F*(1, 29) = 4.12, *p* = .052, ${\upeta }_{p}^{2}$ = .12, depicting a tendency for larger IOR in the second (RR: 347 ms; RC: 331 ms) compared to the first (RR: 360 ms; RC: 349 ms) block. However, block did not modulate any other effects (all *F* ≤ 1.05, all *p* ≥ .313). For overall error rates, participants made descriptively slightly more errors in the second (0.47%) compared to the first (0.23%) block; the said comparison approached but did not reach significance when both values were tested against each other, *t*(29) = 1.75, *p* = .091, *d* = .32.

### Between-experiment comparison

To compare task performance, we added task (discrimination vs. localization) as a between-subjects factor to the 2 (response: repetition vs. change) x 2 (prime valence mapping: positive vs. negative) repeated-measures ANOVA on probe reaction times. There was a main effect of task, *F*(1, 58) = 90.50, *p* < .001, ${\upeta }_{p}^{2}$ = .61, in that participants responded faster in the localization task (347 ms) compared to the discrimination task (514 ms). The significant main effect of response relation, *F*(1, 58) = 16.90, *p* < .001, ${\upeta }_{p}^{2}$ = .23, was further modulated by task,* F*(1, 58) = 67.56, *p* < .001, ${\upeta }_{p}^{2}$ = .54: Whereas in the discrimination task there was a benefit of response repetition (493 ms) over change (534 ms), we found IOR in the localization task, that is, a benefit for response changes (340 ms) over repetitions (353 ms). Neither the main effect of prime valence mapping, *F*(1, 58) = 1.57, *p* = .216, ${\upeta }_{p}^{2}$ = .03, nor its modulation by task, *F*(1, 58) = 0.47, *p* = .496, ${\upeta }_{p}^{2}$ = .01, were significant. Crucially, whereas the two-way interaction of response relation and prime valence mapping did not reach significance, *F*(1, 58) = 3.53, *p* = .065, ${\upeta }_{p}^{2}$ = .06, the three-way interaction of response relation and prime valence mapping by task did, *F*(1, 58) = 6.16, *p* = .016, ${\upeta }_{p}^{2}$ = .10. Thus, the interaction of response relation and prime valence mapping only occurred with discrimination performance and differed significantly from that in localization performance. To pinpoint this, we tested the differential value of the localization task against that of the discrimination task; this *t*-test resembles the between-experiment three-way interaction and was significant, *t*(58) = 2.48, *p* = .016, *d* = 0.64, coming with a *BF*_10_ = 3.26 in favor of the alternative hypothesis given the data.

### Reaction time distributional analysis (explorative analysis)

In the current study, localization task performance was on average 167 ms faster than discrimination task performance. It has been argued (Frings & Moeller, 2012) and empirically observed (e.g., Schöpper et al., 2022b) that retrieval-based effects need time to unfold, which was previously argued to be a possible reason why no S-R binding is found in detection (Schöpper, Hilchey et al., 2020) and localization (Schöpper et al., 2022a; Schöpper & Frings, 2022) performance: According to this view, detection and localization performance is simply too fast to be affected by retrieval. In fact, retrieval-like processes *can* have an impact on late detection (Chao et al., 2020) and localization (Schöpper & Frings, 2022; Schöpper et al., 2022a) responses, which, however, manifests in *benefits* for partial repetitions, often marked by a feature change benefit at location repetitions discussed in the context of non-spatial IOR (e.g., Hu et al., 2011, 2013). However, average responding in detection and localization tasks might be simply so fast, that retrieval-processes – may they spur on effects attributed to S-R binding (e.g., partial repetition costs) or non-spatial IOR-processes (e.g., partial repetition benefits) – are too slow to affect said task performance – which would be a complementary explanation of an absent modulation of IOR by affective information. To test for this, we calculated cumulative reaction time distributions based on reaction time percentiles (e.g., Schöpper & Frings, 2022, 2023; Schöpper et al., 2022a, b; Taylor & Ivanoff, 2005). If overall response speed should be the explanation for the absent interaction, said interaction should emerge at late responses.

### Discrimination task

**Fig. 3:**
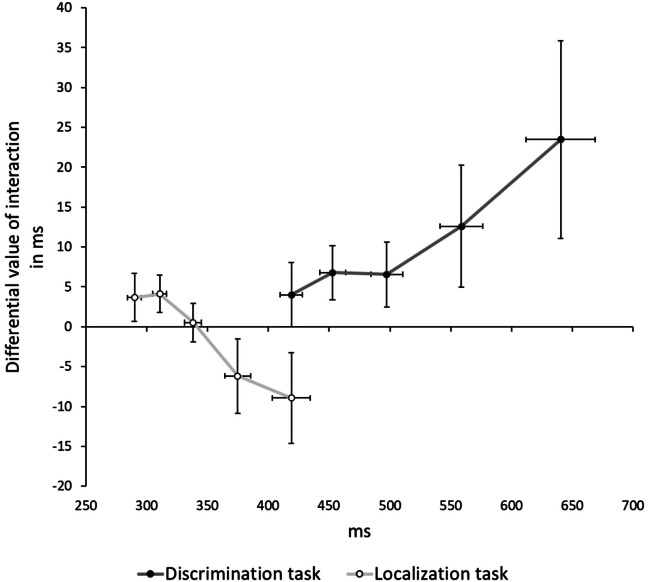
The calculated differential values of the interactions of response relation and valence mapping on the y-axis in ms and reaction times on the x-axis in ms as a function of percentile (cf. delta plot; De Jong et al., 1994; Ridderinkhof, 2002) and experiment. See main text for explanations. The black (Discrimination task) and white (Localization task) dots represent the 10th, 25th, 50th, 75th, and 90th percentile for each function. Error bars represent standard error of each mean of each averaged percentile for the effect of interest (y-axis) and overall reaction time (x-axis)

After applying the cut-off criteria mentioned above, we took the 10th, 25th, 50th, 75th, and 90th percentile of probe reaction times separate for each participant for each condition. We then calculated the differential value of the interaction, (RRN-RRP)-(RCN-RCP), separate for each percentile. A repeated-measures MANOVA with percentile (10th vs. 25th vs. 50th vs. 75th vs. 90th) as the only factor on the calculated differential values revealed no effect, *F*(4, 26) = 0.53, *p* = .717, ${\upeta }_{p}^{2}$ = .08, suggesting that the found interaction did not significantly increase with increasing percentile (10th: 4 ms; 25th: 7 ms; 50th: 7 ms; 75th: 13 ms; 90th: 23 ms; see Fig. 3). The linear trend of the percentile factor was not significant, *F*(1, 29) = 2.06, *p* = .162, ${\upeta }_{p}^{2}$ = .07.

### Localization task

We recalculated the interaction as reported for the discrimination task and performed a repeated-measures MANOVA with percentile (10th vs. 25th vs. 50th vs. 75th vs. 90th) as the only factor on the calculated differential values. The main effect of percentile did not turn significant, *F*(4, 26) = 1.90, *p* = .140, ${\upeta }_{p}^{2}$ = .23, suggesting that the found interaction did not significantly^3^ increase with increasing percentile (10th: 4 ms; 25th: 4 ms; 50th: 1 ms; 75th: -6 ms; 90th: -9 ms; see Fig. 3). However, the linear trend of the percentile factor was significant, *F*(^1,^ 29) = 4.87, *p* = .035, ${\upeta }_{p}^{2}$ = .14.

## Discussion

Analysis of cumulative reaction time distributions revealed that in the discrimination task, the modulation of response repetitions and changes by previously touching on a positive or negative image was stable across percentiles. In the localization task, the linear trend suggested a potential role of increasing percentile affecting the interaction of response relation and valence. This suggests that the emerging trend at late localization response percentiles would have accentuated given more time (cf. Chao et al., 2020; Schöpper et al., 2022a). However, note that this could result in an interaction that is opposite (RRP > RRN; RCP < RCN) to the distribution emerging in discrimination performance (RRP < RRN; RCP > RCN), sharing similarities with partial repetition *benefits* versus partial repetition *costs* emerging at late responses depending on task type (Schöpper et al., 2022a). However, time distributions of tasks in the current study barely overlapped (see Fig. 3), so the between-experiment comparison has to be interpreted with some caution.

## General discussion

In the current study, participants discriminated (Experiment 1) or localized (Experiment 2) target stimuli appearing in prime-probe sequences. All stimuli were presented on a touchpad, on which participants had to execute their responses by tapping on it. Crucially, during the prime display, participants executed their responses to the target stimulus on task-irrelevant images of fruits or spiders, which were absent during the subsequent probe display. When participants had to discriminate target letters (Experiment 1), previously tapping on a fruit or spider differently affected probe responses: Response repetitions were faster for fruit compared to spider mappings; this benefit did not occur – and was descriptively even slightly reversed – for response changes. Following action control theories on S-R binding (e.g., Frings et al., 2020; Hommel, 2004; Hommel et al., 2001), this suggests that task-irrelevant affective information was integrated with the prime response and interfered with probe response execution due to retrieval of positive or negative information. However, when participants had to localize target dots (Experiment 2), affective information during the prime display did not modulate subsequent probe response executions; in Experiment 2, we only observed overall IOR, marked by a benefit of localization response changes (e.g., Schöpper & Frings, 2022). This replicates findings of IOR being stable and unaffected by affective information (e.g., Berdica et al., 2017; Lange et al., 2008).

The absence of a modulation by valence in the localization task is fully in accordance with recently proposed boundaries of S-R binding approaches (e.g., Hilchey et al., 2018; Huffman et al., 2018; Schöpper & Frings, 2022, 2023; Schöpper, Hilchey et al., 2020; Schöpper et al., 2022a), in that binding effects are absent in localization performance. In that sense, affective information can be seen as some kind of non-spatial feature (see Lavender & Hommel, 2007), which is only retrieved in the case of discrimination performance but not in localization performance (cf. Schöpper & Frings, 2022), suggesting task-dependency in the impact of valence on action. Yet, affective information sticks out from other non-spatial features in that it comes with a certain valence and arousal (e.g., Kuppens et al., 2013). Note that in our design the perceptual features of the non-spatial information (spider or fruit images) were never repeated from prime to probe, suggesting that response repetitions retrieved the *pleasantness* or *aversiveness* of the previous response and not simply pure perceptual components of presented objects (e.g., a spider is perceived as an aversive animal and not simply as a colored shape forming an object). This fits well with the impact of affect on action control processes (e.g., Eder et al., 2015; Giesen & Rothermund, 2011), and shows that valence and/or arousal is bound into an event file and retrieved (cf. Frings et al., 2020).

As arousal – a physiological response to spider images (e.g., Geer, 1966; Kolassa et al., 2005) – has been found to not modulate S-R binding (Giesen & Eder, 2022), the effect of our current study might not have been caused by overall arousal of spider images, but by their valence. Although negative emotions have been linked to enhanced memory performance (Kensinger, 2007, 2009), they also have been found to hamper retrieval of other memorized information (Bisby et al., 2018) and demand high processing priority, in turn, impairing task performance (Meinhardt & Pekrun, 2003; see also Hartikainen et al., 2012). Following that, one might muse that the current interaction of the discrimination task could be interpreted as an overall response repetition benefit being impaired by previously presented negative information compared to positive information.

However, a modulation of response repetitions and changes by previously mapped affective information can be best explained by coupling of task-irrelevant stimulus valence with the prime response that was retrieved due to repeating the response. This is fully in accordance with binding approaches in action control (Hommel, 1998, 2004) and highlights the possibility to differently modulate the processes of binding and retrieval in prime-probe sequences (Frings et al., 2020; Laub et al., 2018). Moreover, the pattern in the discrimination task is congruent with approach-avoidance behavior (e.g., Chen & Bargh, 1999; Lavender & Hommel, 2007; see also Rinck et al., 2021, for such in touchpad responses), especially in the context of binding approaches and effect anticipation (Eder & Hommel, 2013; Eder et al., 2012, 2015): Participants are faster in repeating a response that was previously associated with positive compared to negative value. One has to bear in mind that our effects are not due to binding and retrieval benefiting from overall positive valence (Colzato et al., 2007), as we always showed a positive and a negative image simultaneously (see also Berdica et al., 2018; Schöpper et al., 2023).

Eder et al. (2020) found that response priming is stronger for previously rewarded actions compared to unrewarded actions, whereas there was a tendency for response priming being reduced for previously punished compared to unpunished actions. Assuming that response priming is affected by binding and retrieval (Frings et al., 2020; Henson et al., 2014; see also Eder et al., 2020), one might muse that positive and negative images in our study were perceived as rewarding versus punishing; thus, it remains open if our results were due to response repetitions being facilitated by previously touching a fruit image or suffering from previously touching a spider image. Future studies could, for example, investigate how behavior changes if positive and negative images are not paired with each other, but with a neutral picture, each.

Whereas Singh et al. (2018) found that task-irrelevant valence was bound to a response only when it received some attention, valence in our discrimination task modulated responses albeit no instruction was given to attend to it. In Singh et al. (2018) valence was operationalized by using words with positive and negative connotation. Thus, it is possible that positive and negative *words* are simply not processed as strong as positive and negative *images* (Kensinger & Schacter, 2006; Sutton & Lutz, 2019). In turn, affective images, especially with distressing content (Vernon & Berenbaum, 2002), might be a better candidate than affective words for investigating the impact of task-irrelevant valence on behavior.

When participants localized target dots in Experiment 2, we found no overall modulation by affective state but only IOR. This fits well with previous observations of absent modulation of IOR by valence (Berdica et al., 2017; Lange et al., 2008; Stoyanova et al., 2007). In fact, a modulating role of task relevance has been discussed previously (e.g., Berdica et al., 2018; Silvert & Funes, 2016). However, we can now propose a possible reason for IOR in such tasks often being unaffected: In detection and localization procedures typically used to investigate IOR, non-spatial feature repetitions and changes typically do not affect performance (as proposed by action control theories; for an overview, see Huffman et al., 2018, Schöpper & Frings, 2022). Quite fittingly, if a discriminatory component is introduced (e.g., Berdica et al., 2014, 2017; Silvert & Funes, 2016) a modulation by valence in such experimental designs can be found, suggesting that discrimination spurs on retrieval (Schöpper, Hilchey et al., 2020). However, there have been cases of IOR being modulated by valence in localization performance (e.g., Pan et al., 2017), which is congruent with non-spatial features being bound to localization responses under specific conditions (e.g., Hilchey et al., 2019; Schöpper et al., 2022a). Future studies could investigate if these specific conditions influence affective information to the same degree as other non-spatial features.

When we speak of discrimination versus localization performance, one might see these as categorically different. For example, in a localization task one executes a highly automatized response based on spatially compatibility (cf. Kornblum et al., 1990). In contrast, in a discrimination task one has to execute a post-selective response based on a non-spatial feature (Schöpper et al., 2022a; Schöpper, Hilchey et al., 2020; Zehetleitner et al., 2012). This even applies when responding to arrow pointing directions (Schöpper & Frings, 2022), (cued) words with spatial connotation (i.e., “Left” and “Right” in Chinese, Chao, Hsiao et al., 2022; see also Chao & Hsiao, 2021), or post-selectively processing spatial information (Geissler et al., in press; Hilchey et al., 2020; Schöpper et al., 2022a). However, it is also possible to see both tasks as discrimination performance with a different degree of task difficulty. For example, binding effects are reduced in highly practiced tasks (Fournier et al., 2022), which fits with localization tasks – in which one “discriminates” spatially compatible and highly overlearned spatial correspondence (cf. Kornblum et al., 1990) – being simply highly practiced tasks (see also Geissler et al., in press). Further, it has been argued that it is the lack of attention to non-spatial features that results in the absence of binding and retrieval in detection and localization performance (Huffman et al., 2018, 2020). These findings suggest that the differentiation of letter discrimination and location discrimination is not categorical or in discrete steps, but rather on a continuum: Easily executed tasks do not make use of binding and retrieval, whereas increased task difficulty makes these processes mandatory (Geissler et al., in press). Note that this does not limit the generalizability of the observed results. We used a non-spatial discrimination task and compared it with a spatial discrimination/ localization task: If these task types are used in the standard setting, retrieval-based effects in non-spatial discrimination performance emerge but not in localization performance. However, future research could identify the tipping point when retrieval affects responding, for example, by increasing localization difficulty to match it with non-spatial feature discriminability (e.g., Fitousi, 2016).

As it has previously been shown that retrieval effects or, more general, modulations by non-spatial feature repetitions and changes can take time to unfold (e.g., Chao et al., 2020; Chao & Hsiao, 2021; Chao, Hsiao & Huang, 2022; Frings & Moeller, 2012; Schöpper & Frings, 2022; Schöpper et al., 2022a, b), we looked at slow and fast responses in reaction time distributions. In the discrimination task, a modulation by response speed was absent; yet, for the localization task, the differential value for the interaction became more negative at the later percentiles. In turn, it could be argued that the negative trend in the localization task would have continued given slower responses, which however, would be the opposite of that emerging for the discrimination task. Thus, future studies interested in affective influences on responding might manipulate experimental designs so that responses are slowed down. Further, given the literature of late emerging non-spatial IOR effects (Chao et al., 2020; Schöpper et al., 2022a), researchers investigating affective influences on attention using detection and localization procedures might rather refer to non-spatial feature repetition/change effects discussed in the context of attentional orienting (i.e., non-spatial IOR, e.g., Law et al., 1995; Hu et al., 2011, 2013; or repetition blindness, Fox & De Fockert, 2001) than in action control frameworks. To conclude, response speed might have an effect on the emergence of an influence of task-irrelevant valence on behavior; this adds to other time-dependent observations such as responding in the context of spiders being modulated by stimulus-onset-asynchronies (e.g., Flykt & Bjärtå, 2008), stimulus presentation duration (e.g., Mogg & Bradley, 2006), the time course of the experiment (e.g., Zvielli et al., 2015), or effects of repeated stimulus exposure (e.g., Matthews et al., 2012, 2017; Rowe & Craske, 1998).

Crucially, however, if “standard” localization (and detection) procedures are used, responses are typically very fast, and an interaction should not be expected (cf. Huffman et al., 2018; Schöpper & Frings, 2022). This is either due to absent attention to non-spatial identity (Huffman et al., 2018, 2020), a lack of post-selective processing (Schöpper et al., 2022a), overall response speed (Schöpper, Hilchey et al., 2020; see, however, Schöpper & Frings, 2022, submitted), or a combination of a lack of attention and response speed (cf. Chao, Chen & Kuo, 2022). That being said, it is possible that affective information is indeed bound to the response in the prime display even when localizing the stimulus; however, this binding of localization response and affective information is then simply not retrieved (e.g., due to response speed). Support for this comes from the late-emerging trend for the interaction in the localization task, suggesting that affective information was at least processed to some degree in the prime display. Future research could investigate if and how affective information differently affects the coupling and retrieval of affective information in detection and localization procedures. More generally, from an action control perspective in terms of the BRAC framework (Frings et al., 2020), it is unclear if the lack of a binding pattern was caused by affective information not being bound to the prime response or by affective information not being retrieved by the probe response (cf. Schöpper, Hilchey et al., 2020; Frings et al., 2020).

An explorative analysis of the discrimination task indicated that response changes in the first block led to more errors when previously responding on a fruit image compared to a spider image. One might muse that this disadvantage for response changes following a fruit image was driven by avoidance of the area that was previously associated with a spider; however, in that case this pattern would not be caused by retrieval, as the response changes and nothing spurs on retrieval (see footnote 1). Alternatively, it could be that spider stimuli, which have been found to attract attention (e.g., Lipp & Derakshan, 2005), cause initial vigilance (followed by avoidance) in spider phobics (e.g., Pflugshaupt et al., 2005), and might improve response inhibition (Wilson et al., 2015; see, however, Hartikainen et al., 2012; Lindström & Bohlin, 2012; Wilson et al., 2016), attenuated the cognitive system to focus on the task, leading to less errors in case of response changes. However, this effect then diminished in the second block, which might be attributed to habituation (Matthews et al., 2012, 2017; Rowe & Craske, 1998).

In both the discrimination task and the localization task all targets were presented in the upper screen half whereas affective images were presented in the lower screen half. As we observed an interaction in the discrimination task, it is unlikely that the null result in the localization task resulted from a lack of attention to the lower screen half.

We had participants fill out a spider fear questionnaire (Szymanski, & O'Donohue, 1995, translated by Rinck et al., 2002), which score did not modulate the effects of interest (cf. Schöpper et al., 2023), that is, the interaction of response relation and prime valence mapping. Spiders are perceived as disgusting and aversive even for non-distressed individuals (Vernon & Berenbaum, 2002), suggesting that the observed effect might be due to an evolutionary tendency (cf. Rakison & Derringer, 2008) to perceive spiders as dangerous or harmful (see Gerdes et al., 2009) found in a majority of participants. Alternatively, the absence of a correlation between individual fear of spiders and the differential value of each experiment might have been caused by low reliability of the observed effect. In fact, a low reliability of the effect of interest in experimental designs aiming to find effects at a group level is a known problem when correlating them with interindividual measures, such as, for example, negative priming and survey data (for a discussion, see Frings et al., 2015, p. 1583). To test for this, we took odd and even trials (in the raw data) and calculated the differential value of the interaction separate for both. Then, we measured the split-half reliability by calculating the Spearman-Brown-Coefficient, which was *r*_*s*_ = .141 in Experiment 1 and *r*_*s*_ = -.182 in Experiment 2. Further, differential values of odd and even trials were uncorrelated in Experiment 1 (*r* = .08, *p* = .690) and Experiment 2 (*r* = -.08, *p* = .661). Thus, the absence of a modulating role of individual fear of spiders on each interaction might have hinged, to some degree, on the absent reliability of the effect itself. Note, however, that this does not diminish our found effect, as an effect can be replicated at the group level albeit having a low reliability at the interindividual level (e.g., Frings et al., 2015; Titz et al., 2003).

The current experimental results might be of interest for more clinically applied settings, for example, in therapy of arachnophobia. Experimental or therapeutical designs involving the exposure of spider images (e.g., Leutgeb et al., 2009) or real spiders (e.g., Norberg et al., 2018) might incorporate the results of the current study by conducting different tasks with varying task type when confronting spider stimuli: Task type (localization task: no/lower impact of task-irrelevant affective processing on acute behavior; discrimination task: higher impact of task-irrelevant affective processing on acute behavior) might modulate the duration or processing of task-irrelevant aversive information like a real spider.

## Conclusion

Participants discriminated target letters or localized target dots on a touchpad. Mapping positive or negative affect to the response affected subsequent responding. However, this pattern was only observed in the discrimination task and not in localization performance (cf. Huffman et al., 2018; Schöpper & Frings, 2022). The results highlight the complex interplay of affective information on different types of actions, suggesting that affective information does not ubiquitously influence all behavior.

## Appendix 1

In our analysis, we deviate from our pre-registered plan in two occasions. First, we preregistered our analysis with an upper cut-off criterium of 3 interquartile range above the third quartile of a participant’s distribution (Tukey, 1977) for reaction time analyses. Second, we pre-registered to only analyze the second half of trials. Combining both criteria – 3 interquartile range and only analyzing the second block – results in a not-significant interaction of response relation and valence, *F*(1, 29) = 2.60, *p* = .118, ${\upeta }_{p}^{2}$ = .08, which, however, shows the same descriptive data pattern (RRP: 492 ms; RRN: 502 ms; RCP: 537 ms; RCN: 533 ms) reported in the main analysis. Regarding the first point, that upper cut-off came with reaction times being allowable of more than 2000 ms, which are untypically huge for comparable tasks (e.g., Schöpper, Hilchey et al., 2020; Schöpper, Singh, & Frings, 2020) and likely resulted from participants failing to correctly press on the touchpad. Accordingly, using this cut-off for all trials (i.e., first and second block), results in the interaction being not significant, *F*(1, 29) = 2.39, *p* = .133, ${\upeta }_{p}^{2}$ = .08 (RRP: 506 ms; RRN: 510 ms; RCP: 548 ms; RCN: 546 ms). To validate that this was due to outlier trials, we conducted the ANOVA by using the median values of each condition without any temporal cut-offs (only criterion: prime and probe response both correct); doing so results in the interaction of response relation and valence becoming significant, *F*(1, 29) = 4.28, *p* = .048, ${\upeta }_{p}^{2}$ = .13 (RRP: 478 ms; RRN: 488 ms; RCP: 528 ms; RCN: 528 ms). To avoid mean values being obscured by heavy outlier trials in late responses, we report the analysis with a more conservative cut-off in the main text, that is, 1.5 interquartile range above the third quartile of a participant’s distribution (Tukey, 1977). Second, in the main analysis, we included all trials to gain more power and a clearer estimate. However, analyzing only the second block (with 1.5 interquartile range), the interaction on probe reaction times was significant as well, *F*(1, 29) = 4.64, *p* = .040, ${\upeta }_{p}^{2}$ = .14 (RRP: 483 ms; RRN: 493 ms; RCP: 528 ms; RCN: 524 ms), and – as in the main analysis – was not found in probe error rates *F*(1, 29) = 2.49, *p* = .125, ${\upeta }_{p}^{2}$ = .08. We thus conclude that a more conservative cut-off reveals the data pattern better, especially when looking across both blocks.

## Footnotes

1 Alternatively, a response change could be beneficial after previously responding on a spider image, as the location is associated with an aversive stimulus, making a response change beneficial to avoid the said area. However, this would not be caused by retrieval as the response changes and nothing is retrieved.

2 Image IDs in Dan-Glauser and Scherer (2011): Sp008, Sp002, Sp003, Sp004, Sp005, Sp006, Sp007, Sp009, Sp010, Sp011, Sp012, Sp014, Sp020, Sp023, Sp028, Sp033, Sp052, Sp054, Sp058, Sp061, Sp062, Sp063, Sp065, Sp068, Sp072, Sp078, Sp095, Sp097, Sp100, Sp102, Sp103, Sp106, Sp107, Sp109, Sp111, Sp112, Sp116, Sp117, Sp123, Sp124, Sp125, Sp129, Sp130, Sp136, Sp139, Sp143, Sp145, Sp146, Sp149, Sp154, Sp155, Sp158, Sp160.

3 Conducting an ANOVA with Greenhouse-Geisser correction due to violated sphericity, the threeway interaction reached significance, *F*(2.135, 61.92) = 3.11, *p* = .049, ${\upeta }_{p}^{2}$ = .10. However, in the main text we report the MANOVA as it is unaffected by violations of sphericity.
